# Hypertension in Childhood Cancer Survivors: Causes, Screening, and Management

**DOI:** 10.1007/s11906-025-01330-x

**Published:** 2025-03-14

**Authors:** Natalie L. Wu, Coral D. Hanevold

**Affiliations:** 1 Division of Pediatric Oncology, University of California San Francisco Benioff Children’s Hospitals, Oakland, CA USA; 2 Department of Pediatrics, University of Washington School of Medicine, Seattle, WA USA

**Keywords:** Hypertension, Childhood, Cancer, Survivor

## Abstract

**Purpose of Review:**

Survivors of childhood cancer and hematopoietic cell transplant are at risk for developing chronic health conditions, including hypertension. Studies have identified hypertension as an influential risk factor for late kidney dysfunction and cardiovascular disease in childhood cancer survivors. The overall risk of hypertension depends on the specific cancer treatment, from chemotherapy to surgery to radiation. In this report, we aim to review the main causes of hypertension in childhood cancer survivors, with a focus on newer therapies, as well as the current recommendations for screening and management of hypertension in this patient population.

**Recent Findings:**

Novel targeted therapies and immunotherapies are being increasingly used in pediatric cancer treatment, with unclear impact on long-term health. Screening guidelines for hypertension in the survivor population have been issued by various childhood cancer cooperative groups based on best available evidence and expert opinion. Newer studies have focused on individual risk prediction, which may help improve the diagnosis and management of hypertension, particularly in higher-risk individuals.

**Summary:**

Despite the importance of hypertension as one of the few modifiable risk factors for cardiovascular and renal health, studies have yet to define optimal blood pressure targets, screening parameters, or management strategies in childhood cancer survivors. Additionally, further studies are needed to demonstrate improvement in outcomes following interventions for hypertension specifically in this patient population.

## Introduction

Survivors of childhood cancer and hematopoietic cell transplant (HCT) are at risk for developing chronic conditions following treatment [1]. Recent studies in large cohorts of childhood cancer survivors demonstrated a high burden of multiple health events [2, 3]. Specifically regarding kidney toxicities, both acute kidney injury (AKI) and chronic kidney disease (CKD) are relatively common [4–7]. Use of nephrotoxic chemotherapy, irradiation, and surgery such as nephrectomy can all contribute to kidney injury in these patients [8, 9]. Survivors also have a significantly increased risk of cardiovascular disease compared with the general population, representing one of the leading causes of late morbidity/mortality [10, 11]. In the latest statement from the American Heart Association, cancer therapy automatically confers the designation of “At Risk” for cardiovascular disease, while stem cell transplant recipients are considered “High Risk” [12].

Hypertension represents one of the few modifiable risk factors in the development of both CKD [13] and cardiovascular disease, including ischemic heart disease and cardiomyopathy [14]. Therapy contributes to hypertension due to effects on endothelial function, sympathetic activity, and the renin-angiotensin system, in addition to direct nephrotoxicity [15]. Consequently, survivors have been shown to have higher fat mass, greater insulin resistance, and increased arterial stiffness compared with controls [10]. Appropriate management of hypertension then becomes especially critical, as hypertension significantly increases the risk of coronary artery disease, valvular disease, and heart failure, and is independently associated with cardiac death in childhood cancer survivors [16].

In this report, we aim to review the causes of hypertension in childhood cancer survivors, with a focus on newer targeted therapies and immunotherapies that are being increasingly used. We also discuss current screening guidelines for hypertension in the survivor population and the identification and risk classification of risk factors, including recent studies on individual risk prediction. Finally, we comment on current management and interventions for hypertension.

## Epidemiology

Hypertension is one of the more common modifiable cardiovascular risk factors, which also includes diabetes mellitus, dyslipidemia, and obesity [16, 17]. Using data from the Childhood Cancer Survivor Study (CCSS) cohort with > 10,000 survivors, hypertension was self-reported by 14.9% of survivors (at a median age of 33.7 years) and the prevalence only increased with age, with 40.2% reporting hypertension by age 50 years [16]. In another study of > 3,000 adult survivors of childhood cancer from the St. Jude Lifetime Cohort Study (SJLIFE), the cumulative prevalence of hypertension based on in-person blood pressure measurement increased with age and exceeded 70% at age 50 years [17]. Among pediatric and adolescent HCT survivors, prevalence of hypertension was reported as 17% overall at median follow-up of 16 years post-HCT, with 3 × higher prevalence among those 11–17 years old and 2 × higher prevalence among those 18–39 years old compared with age-matched general U.S. population cohorts [18].

Hypertension is more common in certain diagnoses such as Wilms tumor who typically undergo nephrectomy, but the prevalence of hypertension for survivors of all diagnosis groups is higher than expected in the general population, even after accounting for age, sex, race/ethnicity, and BMI category [17]. Within the SJLIFE cohort, 51.4% of Wilms tumor survivors diagnosed with hypertension, with 19.2% meeting criteria for pharmacologic treatment at a median age of 30.5 years [19].

## Etiologies of Hypertension in Childhood Cancer Survivors

### Traditional Treatment-Related Exposures

Various treatment-related exposures can contribute directly to nephrotoxicity and subsequent CKD, which can manifest as hypertension and proteinuria, in addition to decreased kidney function. These exposures can include surgery, radiation, and chemotherapeutic agents. Nephrectomy is a well-described cause of kidney dysfunction, related to inadequate kidney function due to glomerular volume loss [20–22] and compensatory hypertrophy and hyperfiltration injury in the remaining kidney [23, 24]. In multivariate analysis, nephrectomy is significantly associated with later development of hypertension [17]. Abdominal radiotherapy was inconsistently associated with subsequent hypertension in a recent Cochrane review [25]. Radiation nephropathy can manifest as hypertension, proteinuria, or kidney insufficiency [25, 26] with higher risk for CKD in patients who received higher radiation doses [26, 27]. Mechanistically, radiation to the kidney directly damages the glomeruli, tubules, and vascular endothelium [28], with increased risk in patients with history of nephrectomy [21, 29].

Hypertension appears to potentiate cardiovascular damage, particularly among survivors who received chest-directed radiotherapy or anthracycline chemotherapy [16]. Within the cardiovascular system, prior cancer therapy appears to contribute to hypertension via effects on endothelial function, sympathetic activity, and the renin-angiotensin system [15]. Additionally, radiation to the chest can directly damage the pericardium, myocardium, and coronary vessels through other pathways including fibrosis, inflammation, and oxidative stress [30]. In one cohort study of younger childhood cancer survivors age 9–18 years old, this decline in endothelial function was already evident, as measured by carotid artery stiffness and endothelial-dependent arterial dilation [10]. Among survivors in the Childhood Cancer Survivor Study (CCSS), hypertension combined with chest radiotherapy dramatically increased the risk of coronary artery disease, heart failure, and valvular disease (RR 37.2 [95% CI 22.2–62.3], RR 55.8 [95% CI 35.1–88.7], RR 106.8 [31.1–366.9], respectively) [16]. Similarly, the combination of hypertension and anthracycline chemotherapy profoundly increased the risk of heart failure (RR 85.5 [95% CI 45.2–161.8]) [16].

Other traditional chemotherapy agents have been implicated in long-term kidney dysfunction and subsequently hypertension [25, 26, 31–36]. Cisplatin and carboplatin are platinum agents that form DNA cross-links that subsequently interfere with cell replication. Cumulative cisplatin dose is associated with development of CKD [32, 33], with doses ≥ 200 mg/m^2^ considered high risk per the Children’s Oncology Group (COG) Long-Term Follow-Up guidelines [27]. Carboplatin is typically regarded as less nephrotoxic compared with cisplatin but several studies have reported an association with late nephrotoxicity [34, 35]. Cyclophosphamide and ifosfamide are examples of alkylating agents that inhibit DNA synthesis. Total ifosfamide dose ≥ 60 g/m^2^ is associated with higher risk for CKD [27], likely related to both proximal tubular dysfunction and glomerular impairment [37]. Anthracyclines, including doxorubicin and daunorubicin, are intercalating agents that disrupt DNA replication. These agents are well-described to cause cardiotoxicity in a dose-dependent manner, with doxorubicin isotoxic equivalents ≥ 250 mg/m^2^ associated with higher risk for cardiomyopathy, congestive heart failure, and arrhythmias [27]. The direct association of hypertension with specific chemotherapy agents has been harder to elucidate. Among survivors in the SJLIFE cohort, specific treatment exposures were not significantly associated with hypertension aside from low-dose cyclophosphamide exposure, which was felt unlikely to be truly significant [17]. From the Dutch cohort of childhood cancer survivors, treatment with cisplatin or cyclophosphamide was suggestive of increased risk of hypertension (OR 4.3 and 2.1, respectively), although the associations were not statistically significant [36]. Of note, these studies were conducted in relatively young survivors; with longer follow-up, it is possible that the risk of treatment-related hypertension will increase.

### Targeted Therapies and Immunotherapies

Novel targeted therapies are increasingly used in conjunction with and in place of traditional chemotherapy in pediatric cancers (Table 1). Several classes of these newer agents, including tyrosine kinase inhibitors (TKIs) and other small molecule inhibitors, proteosome inhibitors, and cancer immunotherapy have been associated with cardiovascular toxicity and/or nephrotoxicity [4, 30, 38–41]. These described toxicities are generally acute or short-term adverse effects studied in the setting of clinical trials [42]. More studies are needed to describe the long-term cardiovascular and kidney toxicities, including the risk of late hypertension.

**Table 1:** Newer drug classes used in pediatric oncology associated with hypertension

Drug Class	Specific agents	Pathophysiology
Tyrosine kinase inhibitors (TKIs)	Imatinib, dastinib, pazopanib, sorafenib, cabozantinib, lenvatinib	Inhibition of vascular endothelial growth factor signaling pathways leading to endothelial dysfunction, reduced prostacyclin and nitric oxide production, and increased vascular resistance
Serine-threonine-protein kinase B-raf (BRAF) inhibitors	Dabrafenib, vemurafenib, tovorafenib	Reduction in nitric oxide bioavailability leading to vasoconstriction
Mitogen-activated kinase (MEK) inhibitors	Trametinib, cobimetinib	Reduction in nitric oxide bioavailability leading to vasoconstriction
Phosphatidylinositol 3-kinasec (PI3K) inhibitors	Copanlisib	Inhibition of PI3K receptors on endothelial cells, causing vasoconstriction
Proteosome inhibitors	Bortezomib, carfilzomib, ixazomib	Inhibition of antioxidant pathways causing increased oxidative stress, reduced nitric oxide bioavailability, and endothelial dysfunction

TKIs, including imatinib, pazopanib, sorafenib, and cabozantinib, are used in a variety of both solid and liquid malignancies. In general, TKIs increase the risk of cardiovascular toxicity due to anti-angiogenic effects and endothelial dysfunction leading to increased vascular resistance [15, 43]. In a recent meta-analysis, TKIs were significantly associated with acute therapy-related hypertension (RR 3.78 [95% CI 3.15–4.54]) compared with traditional chemotherapy [44], while in another meta-analysis of 77 Phase III trials, TKIs were associated with higher risk of hypertension, cardiac ischemia, arterial thromboembolism, and cardiac dysfunction [43]. These agents can also cause glomerulosclerosis as well as thrombotic microangiopathy within the kidney, although the nephrotoxicity is generally reversible after discontinuation of therapy [38]. Within the category of kinase inhibitors, vascular endothelial growth factor (VEGF) inhibitors, including the monoclonal antibody bevacizumab, are commonly associated with hypertension [15, 42]. In a meta-analysis of 20 randomized controlled studies including over 12,000 cancer patients, the overall incidence of hypertension observed during the study period in patients receiving bevacizumab was 23.6% [45].

Other small molecule inhibitors besides TKIs are emerging as more mainstream therapeutic agents. Serine-threonine-protein kinase B-raf (BRAF) inhibitors, including dabrafenib and vemurafenib, as well as mitogen-activated kinase (MEK) inhibitors, including trametinib and cobimetinib, work by targeting the RAS/RAF/MEK/ERK signaling pathway. These agents are frequently utilized in brain tumors but are also active against melanoma and Langerhans cell histiocytosis. Both BRAF and MEK inhibitors are associated with cardiovascular toxicity, including hypertension, ventricular dysfunction, and arrythmias [46]. In a recent review article, hypertension was noted to be the most common adverse event associated with BRAF/MEK inhibitors, with higher incidence seen in combination therapy [46]. Hypertension due to BRAF/MEK inhibitors is likely the result of reduced nitric oxide availability with subsequent vasoconstriction [41]. Phosphatidylinositol 3-kinasec (PI3K) inhibitors, such as copanlisib, have been studied in both lymphoma and relapsed/refractory solid tumors. They function by targeting the PI3K/AKT/mTOR signaling pathway, Copanlisib is commonly associated with treatment-related hypertension [47], presumably related to inhibition of PI3K receptors on endothelial cells, causing vasoconstriction [48].

Proteosome inhibitors, including bortezomib, carfilzomib, and ixazomib, have classically been used in multiple myeloma and other plasma cell neoplasms, but are currently also being tested in the relapsed leukemia setting. These agents are associated with cardiovascular toxicity, as well as AKI [47], likely related to endothelial dysfunction in the kidneys and/or oxidative stress leading to myocardium and vascular changes [41, 49]. In a recent meta-analysis, hypertension was the most common cardiovascular adverse event in all clinical trials involving carfilzomib, with an estimated incidence of 4.3% (95% CI 2.6–6.4%) for grade ≥ 3 hypertension, although notably these studies were conducted in adults [49].

Cancer immunotherapy is a developing category of therapy that harnesses the host’s immune response. Immune checkpoint inhibitors are generally monoclonal antibodies that function by blocking inhibitory signaling that can cause T cell downregulation, leading to immune system activation. These include cytotoxic T-lymphocyte antigen-4–blocking (CTLA-4) antibodies, including ipilimumab, and programmed cell death protein-1 (PD-1) inhibitors, including pembrolizumab. The adverse effects of these agents are generally immune-mediated, due to the disruption in the immune system between self-tolerance and autoimmunity, with side effects including tubulointerstitial nephritis, glomerulonephritis, and myocarditis [30, 38]. Chimeric antigen receptor-T cells (CAR-T cells) involve re-engineering a patient’s endogenous T-cells to target certain markers on cancer cells, including CD19 and CD22. Complications following CAR-T cell infusion can include acute changes such as cytokine release syndrome and acute kidney injury, which can cause transient blood pressure changes, as well as longer-term side effects such as cardiac dysfunction [30, 50].

### Treatment-Related Complications

Common therapies utilized during cancer treatment or stem cell transplant often contribute to cardiovascular or kidney injury that may lead to hypertension. Antimicrobials such as vancomycin, amphotericin, acyclovir, and cidofovir are all known to be potentially nephrotoxic and can lead to chronic kidney injury. Calcineurin inhibitors such as cyclosporine and tacrolimus are used post-HCT for graft-versus-host-disease (GVHD) prophylaxis. Both agents have been associated with acute kidney injury, chronic kidney disease, and hypertension [28, 51]. Acute elevations in blood pressure post-HCT can be associated with transplant-associated thrombotic microangiopathy (TMA), which results following endothelial damage and activation of the coagulation/complement systems [52], leading to microangiopathic hemolytic anemia and thrombocytopenia, hypertension, hematuria, proteinuria, and AKI [31].

Pediatric cancer patients are also at high-risk for development of sepsis and bacteremia due to immunosuppression, as well as fluid overload from iatrogenic fluid resuscitation and third spacing, both of which can contribute to AKI and subsequent hypertension. Sinusoidal obstructive syndrome (SOS), formerly known as veno-occlusive disease or VOD, is another complication of HCT conditioning regimens [52]. Injury to the liver sinusoidal endothelial cells can lead to hepatic necrosis/fibrosis and vascular obstruction, causing portal hypertension and decreased kidney perfusion, leading to AKI with tubular injury [52, 53]. In terms of later outcomes, previous AKI is a well-known risk factor for subsequent CKD among cancer and HCT survivors [5, 40]. In a study of over 1000 pediatric cancer survivors, recurrent AKI (≥ 4 episodes) was a significant risk factor for impaired renal function (defined as eGFR < 90 mL/min/1.73m^2^) at a median of 5 years after diagnosis [5]. More generally, among all hospitalized children in a large Canadian study, pediatric AKI survivors were at higher risk of developing long-term kidney failure and CKD, as well as hypertension (HR 2.3, 95% CI 2.1–2.6) compared with a matched non-AKI cohort [54].

## Management of Hypertension

### Current Screening Guidelines

Multiple groups have developed guidelines for management of long-term follow-up care for survivors of childhood cancer, including the Children’s Oncology Group (COG) [27], the Dutch Childhood Oncology Group (DCOG) [55], and the Late Effects Group of the United Kingdom Children’s Cancer and Leukaemia Group (UKCCLG) [56] (Table 2). Recently, the International Late Effects of Childhood Cancer Guideline Harmonization Group (IGHG) was formed to standardize recommendations for survivorship care [57]. Guidelines currently exist for surveillance of cardiomyopathy [58, 59] and coronary artery disease [60], while guidelines for nephrotoxicity are currently in development. Specifically for hypertension screening, most professional organizations such as the American Heart Association [12] and the American Academy of Pediatrics [61] recommend routine screening of blood pressure for cancer survivors.

**Table 2:** Recommendations for hypertension screening in cancer survivors among pediatric cancer cooperative groups

Cooperative group	Patient population	Recommendations for hypertension screening	Additional recommendations
Children’s Oncology Group (COG) [27]	Survivors treated with certain chemotherapy (platinums, ifosfamide, anthracyclines); radiation therapy involving head/check, chest, abdomen; hematopoietic cell transplant; nephrectomy	Annual blood pressure screening	Annual Cr/eGFR and urine testing for proteinuria for patients after nephrectomy
Dutch Childhood Oncology Group (DCOG) [55]	Survivors treated with certain chemotherapy (platinums, ifosfamide, nitrosourea derivatives); immunosuppression (e.g. cyclosporin, tacrolimus, prolonged steroids); radiation therapy involving heart, kidneys, or great vessels, nephrectomy	Blood pressure screening at least every 2 years, at every long-term follow-up visit	Early recognition of other cardiovascular risk factors (e.g. diabetes, dyslipidemia, obesity)
United Kingdom Children’s Cancer and Leukaemia Group (UKCCLG) [56]	Survivors treated with certain chemotherapy (platinums, ifosfamide, nitrosureas, high-dose methotrexate, melphalan, anthracyclines); radiation therapy, nephrectomy	Blood pressure screening at least annually	Urinalysis for proteinuria at least annually if renal risk factors
International Late Effects of Childhood Cancer Guideline Harmonization Group (IGHG) [58, 59]	Survivors treated with anthracycline chemotherapy or chest radiation therapy	No specific blood pressure screening guidelines published	“Regular screening” for hypertension, diabetes, dyslipidemia, and obesity

Screening for hypertension is also recommended for patients with other co-morbid conditions, such as dyslipidemia, diabetes, or metabolic syndrome. Most recent IGHG screening guidelines suggest that the greatest risk for cardiomyopathy and heart failure occurs in survivors who received ≥ 250 mg/m^2^ of doxorubicin-equivalent anthracycline equivalents or ≥ 30 Gy of chest-directed radiotherapy [58], which increases the importance of screening for these modifiable cardiovascular risk factors in preventing symptomatic cardiomyopathy. For patients with previous exposure to nephrotoxic therapies, current COG guidelines recommend annual blood pressure screening and baseline labs including electrolytes and BUN/Creatinine at entry into long-term follow-up [27]. Additional evaluation for proteinuria and kidney function with estimated glomerular filtration rate (eGFR) is recommended for patients who have undergone nephrectomy [27], mostly based on the increased risk of CKD and kidney failure in long-term studies of patients with Wilms tumor, most of whom have received nephrectomy [62]. Additionally, consultation to Nephrology is recommended for survivors with hypertension or evidence of progressive kidney insufficiency [27].

### Identifying Novel Risk Factors and Overall Risk Prediction

More recent studies in childhood cancer survivors have focused on identifying genetic variants and their association with certain late effects, with the goal of identifying those with certain genetic susceptibility that may place them at higher risk for developing poor outcomes. Anthracycline-related cardiomyopathy has been the best studied [1, 63], with several genetic variants reported that are associated with anthracycline cardiomyopathy/cardiotoxicity. However, no recommendations are currently in place for cardiomyopathy surveillance in survivors carrying a genetic variant that increases or decreases the risk of developing cardiomyopathy given current lack of evidence [58].

Various risk calculators have been developed for predicting late outcomes in childhood cancer survivors, including heart failure, myocardial infarction and kidney failure [11, 64, 65]. In these prediction models, hypertension was consistently found to be an influential risk factor for the outcomes of interest. In a recent study, early-onset hypertension (diagnosed within 5 years of cancer diagnosis) was determined to be the most influential predictor for late kidney failure, defined as need for dialysis, kidney transplant, or kidney-related death [65]. Other predictors for late kidney failure included Black race/non-Hispanic ethnicity, younger age at cancer diagnosis, genitourinary anomalies, nephrectomy status, and certain treatment exposures (ifosfamide, platinums, anthracyclines, abdominal/flank radiation) [65]. Likewise, in the prediction model for late cardiovascular events, risk factors included younger age at diagnosis and certain treatment exposures (anthracycline chemotherapy and chest radiation), as well as cardiovascular risk factors of diabetes and dyslipidemia, in addition to hypertension [11]. As one of a few modifiable risk factors, improved awareness of hypertension screening and timely treatment may help mitigate late kidney dysfunction and poor cardiovascular outcomes in survivors, especially for patients with multiple risk factors.

### Interventions for Hypertension

The American Heart Association (AHA) recognizes that hypertension should be managed aggressively in patients at higher risk for cardiovascular disease, including those who received cancer therapy (designated “At Risk”) or stem cell transplants (designated “High Risk”) [12]. In their 2017 guidelines for adults, the AHA recommends that both pharmacologic and nonpharmacologic therapies should be used for patients with blood pressure > 140/90 mmHg, however a lower threshold of > 130/80 should be considered for patients with additional risk factors [66]. The American Academy of Pediatrics (AAP) defines hypertension using thresholds of ≥ 95th percentile in children and ≥ 130/80 mmHg in adolescents ≥ 13 years of age, while elevated BP is defined as ≥ 90th and < 95th percentile in children and 120–120/ < 80 mmHg in adolescents [67]. Additional evaluations including ambulatory blood pressure monitoring (ABPM) and serial echocardiograms can be considered for these patients at higher risk for poor cardiovascular outcomes [12]. ABPM has demonstrated better correlation with target organ damage compared with office BP measurements and provides confirmation of suspected hypertension and exclusion of white coat hypertension [67]. Specifically in childhood cancer survivors, in a small prospective cohort of 32 Wilms tumor survivors, 76% had abnormal ABPM parameters using a cut-off of 90th percentile for age, sex, and height, while 34% were diagnosed with masked hypertension [68].

The AAP recommends lifestyle interventions to prevent the development of hypertension and consideration of lower thresholds for treatment initiation in higher-risk patients, such as those with CKD [67]. Unfortunately, the efficacy of lifestyle counseling remains unclear, with one retrospective study finding that childhood cancer survivors seen in a Preventative Cardiology clinic were less likely to improve their blood pressure during the follow-up period after recommended lifestyle modifications compared with age and sex-matched controls [69]. This suggests that more aggressive approaches to preventing and treating hypertension may be needed among childhood cancer survivors beyond the typical recommendations for the general population. When anti-hypertensive agents are indicated, no single class of drugs are preferred over another, although cancer survivors are usually treated with the same agents as recommended for the general population following established guidelines [15, 41]. Generally speaking, the first choices are typically renin-angiotensin system–blockers or calcium channel blockers, with the former favored if proteinuria or nephrotoxicity are present [15].

Anti-hypertensive therapies are also being studied as interventions to prevent other cardiovascular outcomes. A recent study in childhood cancer survivors found that a low-dose beta blocker was cardioprotective in survivors considered at high-risk for heart failure [70]. In this randomized, double-blinded, placebo-controlled study of 182 childhood cancer survivors who received ≥ 250 mg/m^2^ of anthracycline therapy, survivors who received carvedilol had significantly improved echocardiographic indices (left ventricular end-diastolic diameter and end-systolic wall stress) at 2 years [70]. A prior study also suggested that enalapril prevented decline in cardiac function, particularly among patients who received ≥ 300 mg/m^2^ of anthracycline therapy [71].

## Summary

Survivors of childhood cancer and hematopoietic cell transplant are known to be at increased risk for hypertension, as well as associated cardiovascular and kidney diseases. However, this risk differs depending on malignancy type and therapy received, as well as complications associated with treatment. Additionally, more long-term studies are needed to determine the risk posed by novel targeted therapies and immunotherapies that are increasingly used in cancer treatment. Various cooperative groups have issued recommendations for hypertension screening in childhood cancer survivors based on treatment exposures. Guidelines from the American Heart Association and American Academy of Pediatrics suggest that more aggressive diagnosis and management of hypertension should be considered in higher-risk individuals, although data specifically for cancer survivors are limited.

Hypertension represents one of a few modifiable risk factors for cardiovascular and renal diseases, including cardiomyopathy, coronary artery disease, and chronic kidney disease. Studies have identified hypertension as an influential risk factor for the outcomes of late kidney dysfunction and cardiovascular disease specifically in childhood cancer survivors. However, studies have not defined the optimal blood pressure goals for the diagnosis and treatment of hypertension in this patient population. Furthermore, no clinical trials have been conducted to demonstrate mitigation of these adverse outcomes following interventions for hypertension, such as lifestyle modification or pharmacologic therapy. Meanwhile, more aggressive screening may help identify patients at higher risk for complications, such as screening for proteinuria in patients who have received nephrotoxic chemotherapy, especially at high cumulative doses. Successful management of hypertension in these complex patients will require close multi-disciplinary collaboration between Oncology, Cardiology, Nephrology, primary care physicians, as well as the patients themselves.

## Key References

- Cohen JB, Brown NJ, Brown S-A, Dent S, Van Dorst DC, Herrmann SM, et al. Cancer therapy–related hypertension: a scientific statement from the American Heart Association. Hypertension. 2023;80(3):e46-e57.
  - This paper from the American Heart Association summarizes the impact of cancer therapy on hypertension, with a focus on specific chemotherapy agents and potential mechanisms that lead to hypertension.
- Ehrhardt MJ, Leerink JM, Mulder RL, Mavinkurve-Groothuis A, Kok W, Nohria A, et al. Systematic review and updated recommendations for cardiomyopathy surveillance for survivors of childhood, adolescent, and young adult cancer from the International Late Effects of Childhood Cancer Guideline Harmonization Group. The Lancet Oncology. 2023;24(3):e108-e20.
  - This review from an international consortium of survivorship experts summarizes the most recent recommendations for cardiac surveillance among childhood cancer survivors.
- Armenian SH, Hudson MM, Lindenfeld L, Chen S, Chow EJ, Colan S, et al. Effect of carvedilol versus placebo on cardiac function in anthracycline-exposed survivors of childhood cancer (PREVENT-HF): a randomised, controlled, phase 2b trial. The Lancet Oncology. 2024;25(2):235–45.
  - Findings from this randomized controlled study suggest that low-dose carvedilol reduces the risk of cardiovascular complications in long-term childhood cancer survivors treated with high-dose anthracyclines.

## Declarations

### Integrity of Research and Reporting

The manuscript does not contain clinical studies or patient data.
